# Non‐coding RNAs as key player in cancer diagnosis and treatment

**DOI:** 10.1002/2211-5463.70088

**Published:** 2025-09-01

**Authors:** Marcin Majka

**Affiliations:** ^1^ Department of Transplantation, Institute of Pediatrics Jagiellonian University Krakow Poland

## Abstract

Most of the human genome is transcribed into noncoding RNAs (ncRNAs), which although do not encode proteins, are often involved in key cellular processes. Recent technological advances have accelerated the discovery of ncRNA as well as our understanding of ncRNAs as cancer biomarkers and targets. In this “In the Limelight” special issue of *FEBS Open Bio*, four review articles by leading experts provide an overview of the role of ncRNAs in cancer, from their association to liver cancer to their role as oncogenes in head and neck squamous cell carcinoma (HNSCC).

**Abbreviations**
circRNA: circular RNA
HNSCC: head and neck squamous cell carcinoma
lncRNA: long noncoding RNA
miRNA: microRNA
ncRNA: noncoding RNA
PI3K/AKT: phosphoinositide 3‐kinase/protein kinase B

Recent advances in transcriptomics have revealed that a vast majority of the human genome is transcribed into noncoding RNAs (ncRNAs), many of which play fundamental roles in the regulation of gene expression at the epigenetic, transcriptional, and posttranscriptional levels. Among these, microRNAs (miRNAs), long noncoding RNAs (lncRNAs), and circular RNAs (circRNAs) have emerged as critical modulators of oncogenic and tumor suppressor pathways.

LncRNAs, circRNAs, and RNA–DNA hybrid structures (R‐loops) regulate gene expression, chromatin structure, splicing, and signaling in ways that impact all hallmarks of cancer. In many tumors, these ncRNAs are dysregulated, driving uncontrolled proliferation, metastasis, immune evasion, and genome instability. Recent studies show that tissue‐specific ncRNAs can act as potent tumor suppressors or oncogenes, and their altered levels can serve as biomarkers.

This *FEBS Open Bio* “In the Limelight” special issue brings together four reviews to illuminate the following themes: liver‐specific lncRNAs, R‐loops in cancer, oncogenic lncRNAs in head and neck squamous carcinoma, and circRNAs that regulate cytokine and immune signaling in cancer. Collectively, they highlight both shared and unique mechanisms by which ncRNAs shape tumor biology, underscore new technologies for their study, and point to diagnostic and therapeutic opportunities.

Burenina and coworkers focus on liver‐specific lncRNAs and their association with liver cancers, primarily hepatocellular carcinoma and cholangiocarcinoma [1]. While many known cancer‐associated lncRNAs are universal, this review aimed to highlight lncRNAs with preferential expression in the liver or liver tumors, investigating their dysregulation in liver malignancies, their cellular mechanisms, and their potential as biomarkers for diagnosis and prognosis. The authors emphasize that many liver‐enriched lncRNAs are downregulated in liver cancers, suggesting a role as oncosuppressors and potential therapeutic targets, rather than general diagnostic markers. They also highlight the need for rigorous experimental validation and clarification of potentially contradictory findings in future research, particularly regarding lncRNA expression patterns and functional roles in different liver cancer types and diseases.

A review by Wojcik and coworkers focuses on R‐loops [2]. These three‐stranded nucleic acid structures consisting of an RNA–DNA hybrid and displaced single‐stranded DNA have emerged as important regulators of genome stability. In normal cells, R‐loops play roles in transcription, termination, and DNA repair, but when uncontrolled they cause DNA breaks and mutations. Persistent R‐loops accumulate in many cancers (breast, ovarian, lung, etc.) and interfere with DNA repair while activating oncogenes, thereby driving the genomic instability characteristic of tumors. This review underscores the dual nature of R‐loops as both vital cellular regulators and potential threats to genome integrity. It highlights the critical, multifaceted role of human ribonuclease Dicer in managing R‐loop homeostasis, extending beyond its well‐known function in miRNA biogenesis. The dysregulation of Dicer, often leading to aberrant R‐loop accumulation, is strongly implicated in various cancers, offering a new avenue for understanding cancer biology and exploring R‐loops as diagnostic and prognostic biomarkers, especially in conjunction with extracellular vesicle analysis.

Tran and coworkers examine lncRNAs that act as oncogenes in head and neck squamous cell carcinoma (HNSCC) [3]. HNSCC is a significant global health burden with poor prognosis and limited therapeutic options. LncRNAs, a class of nonprotein‐coding RNA molecules > 200 nucleotides in length, are emerging as crucial regulators in HNSCC pathogenesis. Far from being “junk DNA,” lncRNAs play diverse roles in gene regulation, including chromatin modification, mRNA splicing, and interaction with RNA‐binding proteins. Specific lncRNAs promote key oncogenic processes such as cell proliferation, metastasis, immune evasion, and therapy resistance by interacting with pathways such as phosphoinositide 3‐kinase/protein kinase B (PI3K/AKT) and Wnt/β‐catenin pathways. Their dysregulation in HNSCC makes them promising candidates for novel therapeutic strategies and biomarkers for personalized cancer treatment. While significant challenges exist in therapeutic targeting (e.g., delivery, stability, and specificity), ongoing clinical trials highlight their growing clinical significance. LncRNAs represent a frontier in cancer research, offering potential to serve as biomarkers for cancer prediction and prognosis and as promising targets for cancer therapeutics. A deeper understanding of their regulatory mechanisms is crucial for overcoming current therapeutic challenges and translating this promising field into effective clinical applications, especially for targeting previously undruggable genes.

Finally, a review by Joshi and coworkers focuses on circRNAs that regulate cytokine signaling and immunity in cancer [4]. CircRNAs often act as miRNA sponges or protein scaffolds, thereby influencing gene networks. Certain circRNAs sequester miRNAs or bind transcription factors to modulate immune‐related genes and checkpoints. This review highlights the critical and multifaceted role of circRNAs in cancer pathogenesis, specifically focusing on their involvement in regulating cytokine signaling. Dysregulation of cytokine signaling is a central feature of cancer development and progression, influencing both protumorigenic and antitumor immune responses. CircRNAs, characterized by their stable, covalently closed structure, act as crucial transcriptional and posttranscriptional regulators. CircRNAs are dynamic and essential regulators of cytokine signaling pathways in cancer, playing a critical role in tumor initiation, progression, metastasis, and immune evasion. Their unique stability, diverse mechanisms of action (especially as miRNA sponges and protein modulators), and aberrant expression in various types of cancers make them highly relevant for oncology. The ability of circRNAs to influence key protumorigenic processes such as epithelial–mesenchymal transition (EMT), angiogenesis, proliferation, and immune escape via modulation of cytokines, chemokines, and growth factors pathways underscores their significance. Continued research into their biogenesis, mechanisms, and specific roles in different cancer types is crucial for developing novel diagnostic tools and therapeutic strategies.

In summary, all four reviews provide a comprehensive overview of ncRNAs in cancer. They reveal that, despite their diversity, ncRNAs converge on key cancer pathways and immune interactions. Many of these noncoding transcripts function as critical regulatory nodes either as miRNA sponges, epigenetic guides, or genome‐structure modulators, and their misexpression can have profound oncogenic or tumor suppressive effects. Advances in sequencing, high‐throughput screening, and RNA structural analysis are accelerating their discovery, while emerging studies validate ncRNAs as biomarkers and targets. We hope this collection highlights the growing understanding of ncRNAs and inspires readers to further explore their potential as diagnostic approaches and therapeutic avenues in oncology.

## Conflict of interest

The author declares no conflict of interest.

## Author contributions

MM wrote the editorial.
